# Estrogen-related genes influence immune cell infiltration and immunotherapy response in Hepatocellular Carcinoma

**DOI:** 10.3389/fimmu.2023.1114717

**Published:** 2023-02-06

**Authors:** Biao Gao, Yafei Wang, Chonghui Li, Shichun Lu

**Affiliations:** ^1^ Nankai University School of Medicine, Nankai University, Tianjin, China; ^2^ Faculty of Hepato-Pancreato-Biliary Surgery, Chinese PLA General Hospital, Beijing, China; ^3^ Institute of Hepatobiliary Surgery, Chinese PLA General Hospital, Beijing, China

**Keywords:** Hepatocellular Cacinoma, immunotherapy, estrogen, MSI (microsatellite instability), TCR (T cell receptor)

## Abstract

**Background:**

Immunotherapy has been the first-line treatment option in advanced Hepatocellular Carcinoma(HCC); but now, there are no established molecular markers that can predict immunotherapy response. Estrogen has a crucial role in the development of a variety of liver illnesses, including liver fibrosis, Nonalcoholic fatty liver disease (NAFLD), and HCC. Nonetheless, the significance of estrogen-related genes in HCC immunotherapy and the underlying molecular mechanisms are not yet fully understood.

**Method:**

In this study, we constructed a novel estrogen-related gene prognostic signature (ERGPS) by analyzing bulk RNA sequencing data from 365 HCC patients. Based on the median risk score, we divided 365 HCC patients into low- and high-risk groups. Tumor mutation burden (TMB), Microsatellite instability (MSI), T cell receptor (TCR) richness, B cell receptor (BCR) richness, single-nucleotide variants (SNV) Neoantigens, Cancer Testicular Antigens (CTA) scores, and Tumour Immune Dysfunction and Exclusion (TIDE) scores were used to evaluate the magnitude of immunotherapy response. Multiple external datasets validate the validity and robustness of the prognostic signature. Real-time quantitative polymerase chain reaction (qRT-PCR) was used to validate estrogen-related gene overexpression in HCC tissue samples.

**Results:**

ERGPS is an independent risk factor affecting the prognosis of HCC patients and is superior to other clinical variables in predicting patient survival and immunotherapy response. Multiple independent external datasets confirmed the superior predictive efficacy of the prognostic signature. The prognostic signature was positively correlated with TMB score, MSI score, TCR richness, BCR richness, SNV Neoantigens score, CTA score, expression levels of immune checkpoint-related genes, and TIDE score. Patients with HCC in the high-risk group identified by the prognostic signature were likely to be more responsive to immunotherapy and more suitable for immunotherapy. qRT-PCR confirmed that estrogen-related genes of the construct signature were highly expressed in HCC tumor tissues.

**Conclusion:**

Estrogen-related genes are overexpressed in HCC tissues. Our novel prognostic signature can accurately predict not only the prognosis but also the immunotherapy response of HCC patients. In the future, prognostic signatures will be a useful tool for clinicians to screen patients with HCC who are suitable for immunotherapy.

## Introduction

Primary liver cancer is the sixth most prevalent malignancy and the third main cause of cancer-related deaths worldwide. Primary liver cancer includes hepatocellular carcinoma (75%-85% of cases), intrahepatic cholangiocarcinoma (10%-15% of cases), and other rare types (1). Due to the insidious onset of the disease, most individuals with liver cancer are already in the advanced stage at the time of diagnosis, and hence lost the time window for radical surgery. In recent years, the breakthrough of immunotherapy research represented by immune checkpoint inhibitors has broken the traditional treatment pattern of liver cancer and opened a new chapter in the treatment of advanced liver cancer (2–5). However, despite the current achievements in immunotherapy for liver cancer, it still faces challenges such as low objective response rates, lack of effective biological markers to predict treatment response, andimmune-related adverse events (irAEs). Meanwhile, only a small percentage of patients benefit from immunotherapy, and even a few patients develop hyperprogressive disease (HPD) after receiving immunotherapy. Therefore, the development of biological markers that can accurately predict the response to immunotherapy and thus assist doctors to screen patients with HCC who are suitable for immunotherapy has become an urgent task.

The tumor microenvironment (TME) plays a vital role in the progression and development of HCC. In addition to tumor cells, the tumor microenvironment of HCC includes stromal cells (such as immune cells, fibroblasts, endothelial cells, etc.), structural components (such as extracellular matrix, etc.), and signaling components (chemokines, cytokines, and growth factors, etc.), which influence tumor immune evasion, response to immunotherapy, and patient prognosis (6–8). As a result, a better understanding of the crosstalk among the components of the HCC immune microenvironment, as well as the identification of potential therapeutic targets from it, can aid in the prediction of immunotherapeutic response, determination of immunotherapeutic efficacy, identification of prognostic markers, and guidance of individualized treatment regimens.

The incidence of HCC shows considerable gender disparities, with a much greater incidence in males than in women, with the ratio usually between 2:1 and 4:1; while female patients have a better prognosis and longer survival time than male patients (9). It is thought that estrogen has a crucial role in the occurrence and progression of HCC (10, 11). Although there is emerging evidence that estrogen is a potentially critical host factor in HCC advancemen, the potential mechanisms of estrogen-related genes in HCC development and immunotherapy are yet unknown.

In this study, to investigate the relationship between estrogen -related genes and HCC, we performed a comprehensive analysis of bulk RNA sequencing data from 365 HCC patients and single-cell sequencing data from 16 HCC patients and constructed a novel prognostic signature based on 3 key estrogen -related genes.TMB, MSI, CTA score, SNV Neoantigens score, mRNA expression levels of immune checkpoint-related genes, and TIDE score were used to evaluate the efficacy of immunotherapy in different risk groups. The prognostic signature showed superiority in predicting both prognosis and response to immunotherapy in HCC patients, and its validity was validated by many independent external data sets. In the future, the prognostic signature will be a useful tool for clinicians to screen populations suitable for immunotherapy.

## Materials and methods

### Data and clinical samples

The bulk RNA sequencing data and clinical information of 424 HCC patients were downloaded from the TCGA database(https://portal.gdc.cancer.gov/), including sequencing information of 365 HCC tissue samples and 59 paracancerous tissue samples as the training set. The bulk RNA sequencing data and clinical information from 231 HCC patients were downloaded from the ICGC database(https://dcc.icgc.org/) as validation sets. As validation sets, sequencing and clinical data from 115 HCC patients were extracted from the GEO database (https://www.ncbi.nlm.nih.gov/geo/). From CellMarker2.0 (http://yikedaxue.slwshop.cn/), 731 estrogen-related genes were extracted. Transcriptomic and matched clinical data were acquired from the IMvigor210 and GSE9106 cohort of anti-PD-L1-treated patients to investigate the predictive utility of ERGPS for immunotherapy response. The clinical features of 365 HCC patients in the training set are shown in **Table 1**. **Figure S1** shows the data analysis process.

**Table 1:** Clinical characteristics of 365 HCC patients in TCGA LIHC.

Characteristics	Samples (n=365)	Percentage (%)
Gender		
Female	120	32.9%
Male	245	67.1%
Age		
>=60	200	54.8%
<60	165	45.2%
Tumor Stage		
Stage I	180	49.3%
Stage II	91	25.0%
Stage III	81	22.2%
Stage IV	13	3.6%
M Stage		
M0	263	72.1%
MX	102	28.0%
N Stage		
N0	248	67.9%
N1	5	1.4%
NX	112	30.7%
Tumor Grade		
G1	58	15.9%
G2	176	48.2%
G3	116	31.8%
G4	13	3.6%

### Identification of differentially expressed Estrogen-related genes

To identify differentially expressed genes, we analyzed the difference between 365 HCC samples and 59 paracancarcinoma tissue samples in the training set using the “limma” package of R software and set the criterion as p-value 0.05 and log(FC) > 1 or log(FC) -1. Subsequently, the intersection of differentially expressed genes and estrogen-related genes yielded differentially expressed estrogen-related genes.

### Construction and validation of a prognostic signature based on estrogen-related genes

We first screened the genes influencing the prognosis of HCC patients in the training set with a p-value of 0.05 using univariate Cox analysis of differentially expressed estrogen-related genes. To eliminate overfitting, a least absolute shrinkage and selection operator (LASSO) Cox regression analysis was employed in conjunction with the “glmnet” package. Finally, Cox multivariate analysis was utilized to screen for independent risk factors affecting the prognosis of HCC patients with screening criterion of p-value < 0.05. Subsequently, we used the selected genes to construct a prognostic signature. Risk score was computed using the following formula: Risk score = (Expi*Coefi). Coefi and Expi respectively represent the risk coefficient and gene expression. According to the median risk score, 365 HCC patients in the training set were divided into high-risk group and low-risk group. The Kaplan-Meier survival curve was used to compare survival differences between the two groups. The receiver operating characteristic curve(ROC) was used to test the predictive power of risk scores for HCC patients. To verify the robustness of the signature, we first used Kaplan-Meier survival analysis to compare survival differences between high-risk and low-risk groups in different clinical subgroups. To validate the efficacy of the prognostic signature, we applied the same algorithm to calculate the risk score of HCC patients in two independent external data sets and utilized the median value of risk groups to classify HCC patients into high-risk and low-risk groups. Kaplan Meier survival analysis was used to compare survival differences between the two groups. ROC curve was used to test the predictive efficacy of prognosis signature for HCC patients.

### Pathways and functional enrichment analysis

In order to further explore the molecular mechanism behind differential genes, the “clusterProfiler” of R software is used for GO function enrichment analysis and KEGG function enrichment analysis. A p-value of less than 0.05 was considered to show substantial enrichment. Software for Gene Set Enrichment Analysis (GSEA) was used to compare the pathway enrichment scores of the high-risk and low-risk groups. The gene expression profile of the two risk samples was used to evaluate the associated pathways and molecular processes, and the reference gene sets “c2.cp.kegg.v7.4.symbols” were obtained from the molecular signatures database(https://www.gsea-msigdb.org/gsea/msigdb).

### Immune cell infiltration analysis and gene sets variation analysis

Based on the principle of linear support vector regression, CIBERSORT deconvolutes the expression matrix of human immune cell subtypes and determines the content of each subtype (12). The R software’s CIBERSORT function is used to deconvolute the bulk RNA sequencing data of 365 HCC patients and to quantify the fraction of immune cells in the tumor microenvironment for each patient in the training set. The difference in the infiltration of 22 types of immune cells between the high-risk and low-risk groups was compared. GSVA (Gene Set Variation Analysis) is a nonparametric and unsupervised analysis method, primarily used to evaluate the microarray and transcriptome gene set enrichment results (13). In addition, the GSVA function of the R software is used to compute the enrichment score for each patient’s 28 types of immune cells. Additionally, we compared the enrichment scores of the two groups.

### Estimation of stromal and immune scores

The ESTIMATE algorithm can estimate the matrix score and immune score of tumor samples according to transcriptome data, which is used to represent the relative proportion of matrix and immune cells (14, 15). Using the R software’s ESTIMATE function, the Stromal score, immunological score, ESTIMATE score, and tumor purity score of each patient in the training set are evaluated. The Wilcoxon t-test was utilized to analyze the difference between the high-risk and low-risk groups in terms of immune cell infiltration score.

### Mutation analysis and microsatellite instability

To examine the difference between the tumor cell mutation gene and TMB between high-risk and low-risk groups, we obtained the mutation data of 365 HCC patients in the training set from the TCGA database and analyzed the mutation data using the “maftools” package (16) of R software. TMB and MSI can be utilized as useful biomarkers of immune checkpoint inhibitors, as demonstrated in numerous solid malignancies (17, 18). To analyze the difference between the high-risk group and the low-risk group in response to immunotherapy, we evaluated the differences in tumor mutation genes, TMB and MSI, between the two groups.

### Prediction and validation of immunotherapy response

Immunotherapy is playing an increasingly important role in the treatment of advanced hepatocellular carcinoma. The mRNA expression levels of immune checkpoint-associated genes are the basis of immunotherapy. To identify the degree of immunotherapy response in different risk groups, we analyzed the expression differences of mRNA of immune checkpoint-related genes in high-risk and low-risk groups. Richness and the Shannon Diversity Index were used to describe the diversity of the TCR repertoire. Richness measures the number of unique TCRs in the sample, while the Shannon diversity index reflects the relative abundance of different TCRs. BCR is a B lymphocyte receptor and BCR richness is a combination of the various BCR isoforms produced in an individual. TCR richness and BCR richness can be utilized to determine the intensity of immunotherapy response, according to previous research. Neoantigens are novel antigens created by tumor cells. The quantity of tumor neoantigens corresponds with the effectiveness of PD-1/PD-L1 antibodies as immunotherapy improves the immune system’s ability to recognize and destroy neoantigens on the surface of tumor cells. The cancer testicular antigens (CTA) score is used to evaluate tumor immunogenicity, which indirectly reflects the intensity of the immunotherapy response. In order to assess disparities in immunotherapy response, we examined the differences in TCR, BCR, SNV, and CTA scores between the high-risk and low-risk groups. Tumor immune dysfunction and rejection (TIDE) consists of two major mechanisms through the expression of characteristic genes: the induction of T cell dysfunction in tumors with high CTL infiltration and the blocking of T cell infiltration in tumors with low CTL infiltration to construct mathematical prediction models that can also serve as new biomarkers to predict the response to immune check inhibitors. We computed TIDE scores for each HCC patient and evaluated the distributional differences between the high-risk and low-risk groups in the training set.

### Independent external immunotherapy data for validation of immunotherapy response

To effectively confirm the differences in immunotherapy response among risk groups, we obtained comprehensive bulk RNA sequencing data and clinical information from two external immunotherapy data cohorts (IMvigor210,GSE9106). We calculated risk scores for patients receiving immunotherapy in both immunotherapy datasets using the same formula as in the training set, and classified patients into high-risk and low-risk groups based on the median risk score. The proportion of patients who achieved CR/PR(CR: complete response; PR: partial response) or SD/PD(SD: stable disease;PD: progressive disease) following immunotherapy was compared between the two groups to identify the differential in response to immunotherapy between risk groups.

### Drug sensitivity prediction

The pRRophetic package of R software was used to predict the sensitivity (IC50 values) of 138 drugs in the GDSC database in combination with model gene expression data, and the sensitivity of HCC patients to drug therapy was assessed by IC50 values. The differences in IC50 values between the risk groups were compared, and the Wilcoxon test was performed to identify drugs with significant differences between the two groups.

### qRT-PCR confirmed the overexpression of three estrogen-related genes in HCC tissues

To evaluate the robustness of the signature in clinical samples, we gathered tumor and paraneoplastic tissue samples from 12 patients with HCC verified by postoperative pathology. We used qRT-PCR to validate the mRNA expression levels of the three estrogen-related genes used to construct the prognostic signature.

### Statistical analysis

Statistical tests were performed by R statistical computing software (R version 4.1.2). Comparisons between two groups were estimated for significance by the nonparametric Wilcoxon test, and in multiple comparisons by the Kruskal-Wallis test. Categorical data were tested by chi-square test and trend chi-square test. K-M analysis of overall survival(OS) between different subgroups was performed followed by log-rank test. Univariate and multivariate Cox regression analyses were used to screen for independent risk factors affecting the prognosis of HCC patients. A significance criterion of P < 0.05 was selected.

## Results

### Identification of differentially expressed estrogen-related genes

**Figure 1:**
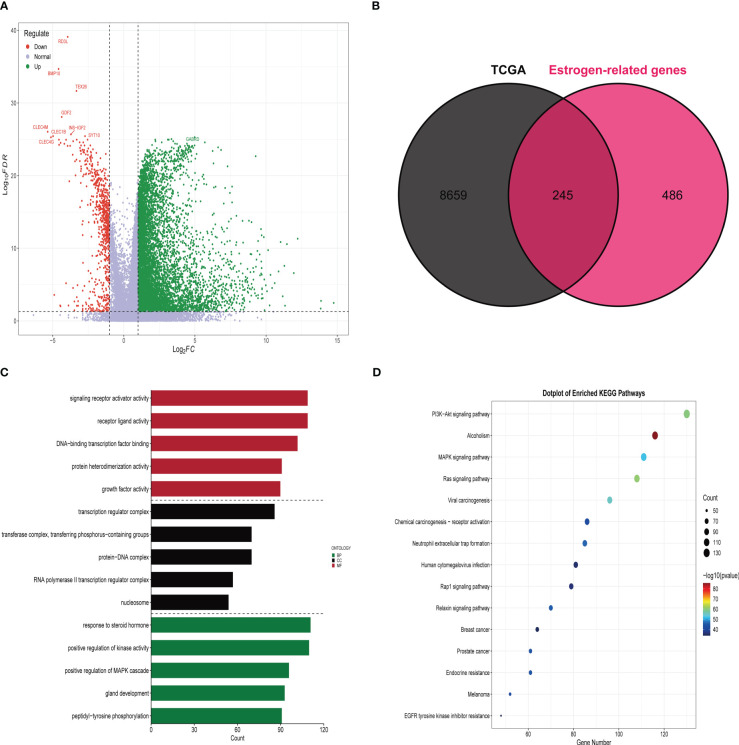
Identification and functional enrichment analysis of differentially expressed estrogen-related genes. **(A)** The volcano map demonstrates that most genes are highly expressed in HCC tissues compared to paraneoplastic tissues. **(B)** Venn diagram demonstrating that there are 245 estrogen-related genes differentially expressed in HCC tissue and paracancerous tissue. **(C)** Results of GO functional enrichment analysis of estrogen-related genes. **(D)** Results of KEGG functional enrichment analysis of estrogen-related genes.

A total of 8904 differential genes were identified by differential analysis of bulk RNA sequencing data from 365 HCC and 59 paraneoplastic tissue samples in the training set, with the set criteria of p-value of < 0.05 and a |log2 (fold change) of |> 1(**Figure 1A**). The intersection of 8904 differential genes with 731 estrogen-related genes resulted in the identification of 245 differential estrogen-related genes (**Figure 1B**). To investigate the signaling pathways associated with differential estrogen-related genes, we did GO and KEGG functional enrichment analysis on these genes. The results of GO functional enrichment analysis showed that these differential estrogen-related genes were mainly enriched based on signaling receptor activator activity, receptor-ligand activity, and DNA (**Figure 1C**). KEGG functional enrichment analysis showed that these differential estrogen-related genes were mainly enriched in PI3K-Akt signaling pathway, MAPK signaling pathway, and Ras signaling pathway(**Figure 1D**). The PI3K-Akt signaling pathway, MAPK signaling pathway, and Ras signaling pathway play important roles in the development and progression of various tumors, and these results suggest that estrogen-related genes may play role in HCC through these signaling pathways.

### Construction and validation of estrogen-related genes prognostic signature

**Figure 2:**
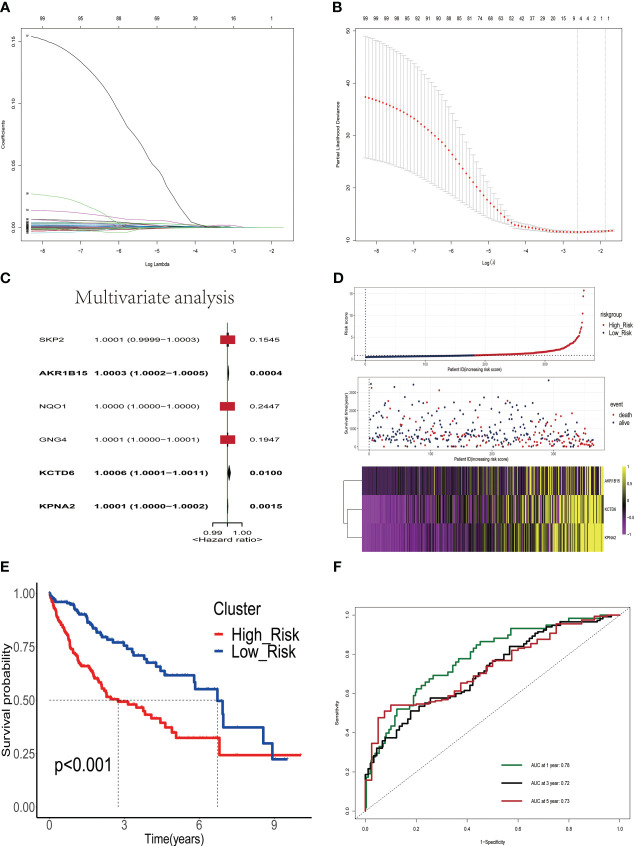
Construction and validation of a prognostic signature based on estrogen-related genes. **(A)** The coefficients of genes calculated by multivariate Cox regression using LASSO. **(B)** The partial likelihood deviance of genes. **(C)** Results of multivariate analysis of six differentially expressed estrogen-related genes. **(D)** The association of risk scores with survival status and gene expression in HCC patients. **(E)** Kaplan-Meier curves were used to compare the overall survival of HCC patients between the high-risk and low-risk groups. **(F)** ROC curves of the prognostic signature for predicting the risk of death at 1, 3, and 5 years.

We analyzed 245 differential estrogen-related genes using univariate Cox regression analysis and screened 99 genes that had an impact on the prognosis of HCC patients with a set criterion of p<0.05. Then, LASSO regression and Cox proportional hazard model studies were utilized to determine the optimal model in the training dataset (**Figures 2A, B**). In addition, multivariate Cox regression analysis was utilized to analyze six differential estrogen-related genes, resulting in the identification of three genes that may serve as independent risk factors influencing the prognosis of HCC patients (**Figure 2C**). We determined each patient’s risk score using the following formula: Risk Score = 3.365e-04*AKR1B15 mRNA expression plus 6.65e-04*KCTD6 mRNA expression plus 1.41e-04*KPNA2 mRNA expression. Based on the median risk score, 365 HCC patients were divided into high-risk group or low-risk group in the training set. As shown in the heatmap, the mRNA expression levels of 3 estrogen-related genes were significantly higher in the high-risk group than in the low-risk group; the mortality rate of patients gradually increased with increasing risk scores(**Figure 2D**). The Kaplan-Meier survival analysis revealed that the overall survival time of patients in the high-risk group in the training set was considerably inferior to that of patients in the low-risk group (**Figure 2E**). To assess the predictive accuracy of the risk signature, time-dependent area under the ROC curves for OS was calculated, and the 1-, 3-, and 5-year AUC values were 0.78, 0.72, and 0.73(**Figure 2F**), respectively.

### Validation of the ERGPS in different clinical subgroups

We first evaluated the relationship between ERGPS and clinical characteristics including age, sex, N stage, M stage, tumor grade, tumor stage, microvascular invasion(MVI), and survival status. Patients with higher tumor grade and tumor stage had higher risk scores. Also, patients who died had a higher risk score compared to those who survived(**Figure S2**). The predictive value of ERGPS was first evaluated in TCGA LIHC patients of different age, gender, microvascular invasion, tumor grade and tumor stage. The results revealed that high-risk score was significantly correlated with an inferior prognosis in the young(P<0.001, **Figure S3A**), old(P<0.001, **Figure S3B**), male(P<0.001, **Figure S3C**) or female(P=0.04, **Figure S3D**), MVI negative(P=0.024, **Figure S3E**), MVI positive(P=0.031, **Figure S3F**) or low tumor grade(P=0.033, **Figure S4A**), high tumor grade (P= 0.007, **Figure S4B**), early stage(P= 0.004, **Figure S4C**) or advanced stage(P= 0.005, **Figure S4D**).

### External validation of the robustness of the ERGPS in two independent cohorts

**Figure 3:**
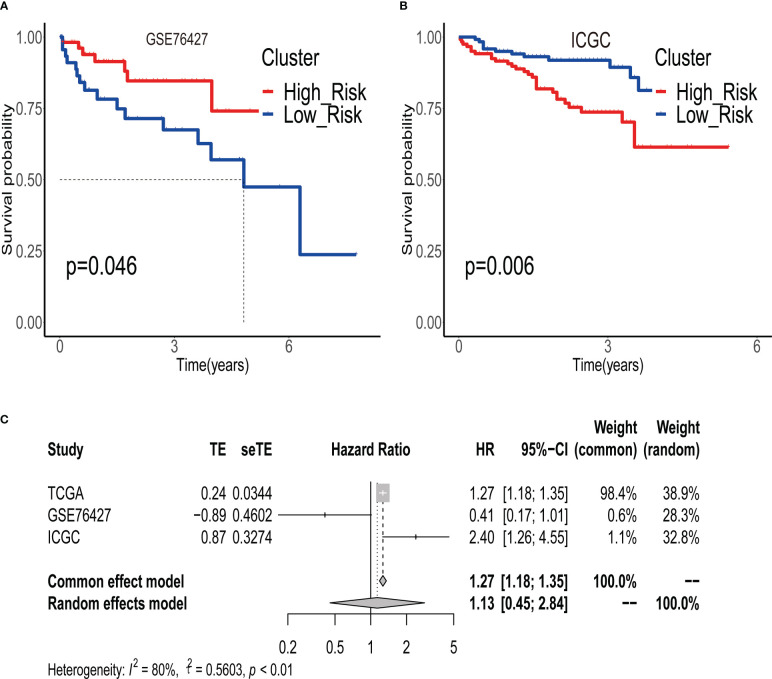
Validity of prognostic signature validated by multiple independent external datasets. **(A)** In the GSE76427 dataset, the overall survival time of patients in the high-risk group group was significantly better than that of patients in the low-risk group. **(B)** In the ICGC dataset, the overall survival time of patients in the high-risk group group was significantly better than that of patients in the low-risk group. **(C)** Results of meta-analysis confirm that prognostic signature is an independent risk factor affecting the prognosis of HCC patients.

To validate the robustness of ERGPS, we included two independent cohorts in this study. We calculated risk scores for each patient in 2 independent cohorts using the same formula and divided HCC patients into high- or low-risk groups based on the median risk score. Kaplan–Meier analysis demonstrated that the high-risk group had an inferior prognosis than the low-risk group in both of these 2 independent cohorts, namely GSE76427 (**Figure 3A**, HR=0.411,95%CI:0.1668-1.0130,p=0.046) and ICGC (**Figure3B**, HR=2.397, 95%CI:1.262-4.555,p=0.00758). In addition, we evaluated the cumulative predictive value of the training set (TCGA) and the two validation sets for the prognosis of HCC patients by conducting a prognostic meta-analysis utilizing the random effects model R software meta. The results of the meta-analysis indicated that ERGPS was an extremely accurate predictor of prognosis for HCC patients (**Figure 3C**, HR = 1.13, 95% CI = 0.45-2.05, p<0.05).

### Identification of ERGPS as an independent prognostic factor

**Figure 4:**
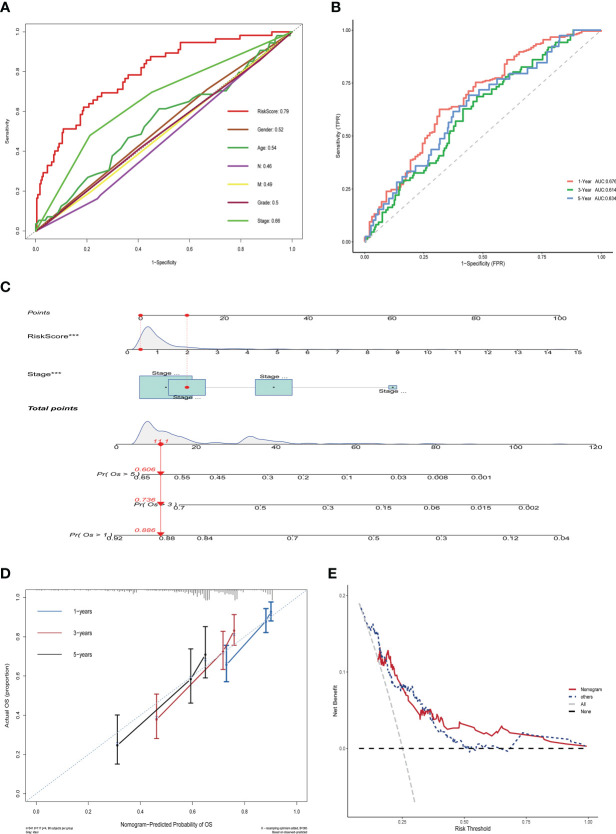
Nomogram construction and validation in TCGA LIHC dataset. **(A)** ROC curves showing the comparative results of risk scores and other clinical factors for prognostic prediction.**(B)** ROC curves showing the results of nomogram for predicting 1-, 3-, and 5-year survival in HCC patients.**(C)** Nomogram constructed from Stage and risk score.**(D)** Calibration curves show strong predictive power of nomogram for 1-, 3-, and 5-year survival in HCC patients. **(E)** Decision curves show that the nomogram model outperforms other models.

To assess the impact of ERGPS on the prognosis of HCC patients, we included clinical characteristics including age, gender, N stage, M stage, tumor grade, and tumor stage in the training set for analysis. Univariate Cox regression analysis showed an effect of tumor stage and risk score on the prognosis of HCC patients (p<0.05). The results of multivariate Cox regression analysis showed that tumor stage and risk score were independent risk factors affecting the prognosis of HCC patients (p<0.05) (**Table 2**). We compared the predictive efficacy of risk scores and clinical characteristics for the prognosis of HCC patients in the training set using ROC curves. The results revealed that the area under the curve for risk scores was greater than that for clinical features (**Figure 4A**), indicating that risk scores are more predictive for HCC patients than clinical characteristics. To forecast the prognosis of HCC patients more intuitively and precisely, we analyzed the tumor grade and risk score using the random forest method and created a nomogram to predict the prognosis of HCC patients (**Figure 4B**). The ROC curve demonstrated the excellent predictive power of the nomogram for overall survival time at 1, 3, and 5 years in HCC patients (**Figure 4C**). Calibration curve indicates the great accuracy of the nomogram in predicting HCC patient survival at 1, 3, and 5 years (**Figure 4D**). The decision curve confirmed that the nomogram, constructed from the tumor stage and risk score, significantly outperformed the other factors (**Figure 4E**).

**Table 2:** Results of univariate and multivariate Cox regression analyses of clinical characteristics and risk scores in the TCGA LIHC dataset.

Characteristics	Univariate analysis		Multivariate analysis	
	Hazard ratio (95% CI)	P value	Hazard ratio (95% CI)	P value
Age	1.011 (0.997-1.026)	0.125		
Gender				
Female	Reference			
Male	0.792 (0.545-1.150)	0.221		
G stage				
G1+G2	Reference			
G3+G4	1.186 (0.819-1.716)	0.366		
M stage				
M0	Reference			
MX	1.392 (0.915-2.117)	0.122		
N stage				
N0	Reference			
NX	1.228 (0.845-1.961)	0.239		
Stage				
StageI+StageII	Reference			
Stage III+Stage IV	2.409 (1.664-3.487)	<0.001	2.268 (1.566- 3.285)	<0.001
RiskScore	1.266 (1.184-1.355)	<0.001	1.268 (1.178-1.363)	<0.001

### Gene set enrichment analysis and functional enrichment analysis

To further examine the molecular mechanism underlying ERGPS, we performed the differential analysis of bulk RNA sequencing data from high-risk and low-risk patients to identify differentially expressed genes, using the following criteria: p-value 0.05, log(FC) > 1 or log(FC) < -1. In total, we obtained 3723 differentially expressed genes, of which 3590 were overexpressed in the high-risk group and 133 in the low-risk group (**Figure S5A**). GO functional enrichment analysis of overexpressed genes in the two groups showed that the overexpressed genes in the high-risk group were mainly enriched in the passive transmembrane transporter activity, channel activity, and ion channel activity signaling pathways(**Figure S5B**), while the low-risk group was mainly enriched in the tetrapyrrole binding, heme binding and receptor-ligand activity signaling pathways(**Figure S5C**). GSEA enrichment analysis showed that the high-risk group was mainly enriched in the KEGG_CELL_CYCLE, KEGG_RNA_DEGRADATION, and KEGG_OOCYTE_MEIOSIS signaling pathways, while the low-risk group was mainly enriched in the KEGG_PRIMARY_BILE_ACID_BIOSYNTHESIS, KEGG_COMPLEMENT_AND_COAGULATION_CASCADES, and KEGG_LINOLEIC_ACID_METABOLISM signaling pathways(**Figure S5D**). The enrichment results of the HALLMARK pathway showed that E2F_TARGETS, G2M_CHECKPOINT, and DNA_REPAIR were mainly enriched in high-risk group, while COAGULATION was mainly enriched in low-risk group(**Figures S6A–D**), suggesting that estrogen in tumor microenvironment was involved in the regulation of cell cycle.

### Association between ERGPS and immune cell infiltration

**Figure 5:**
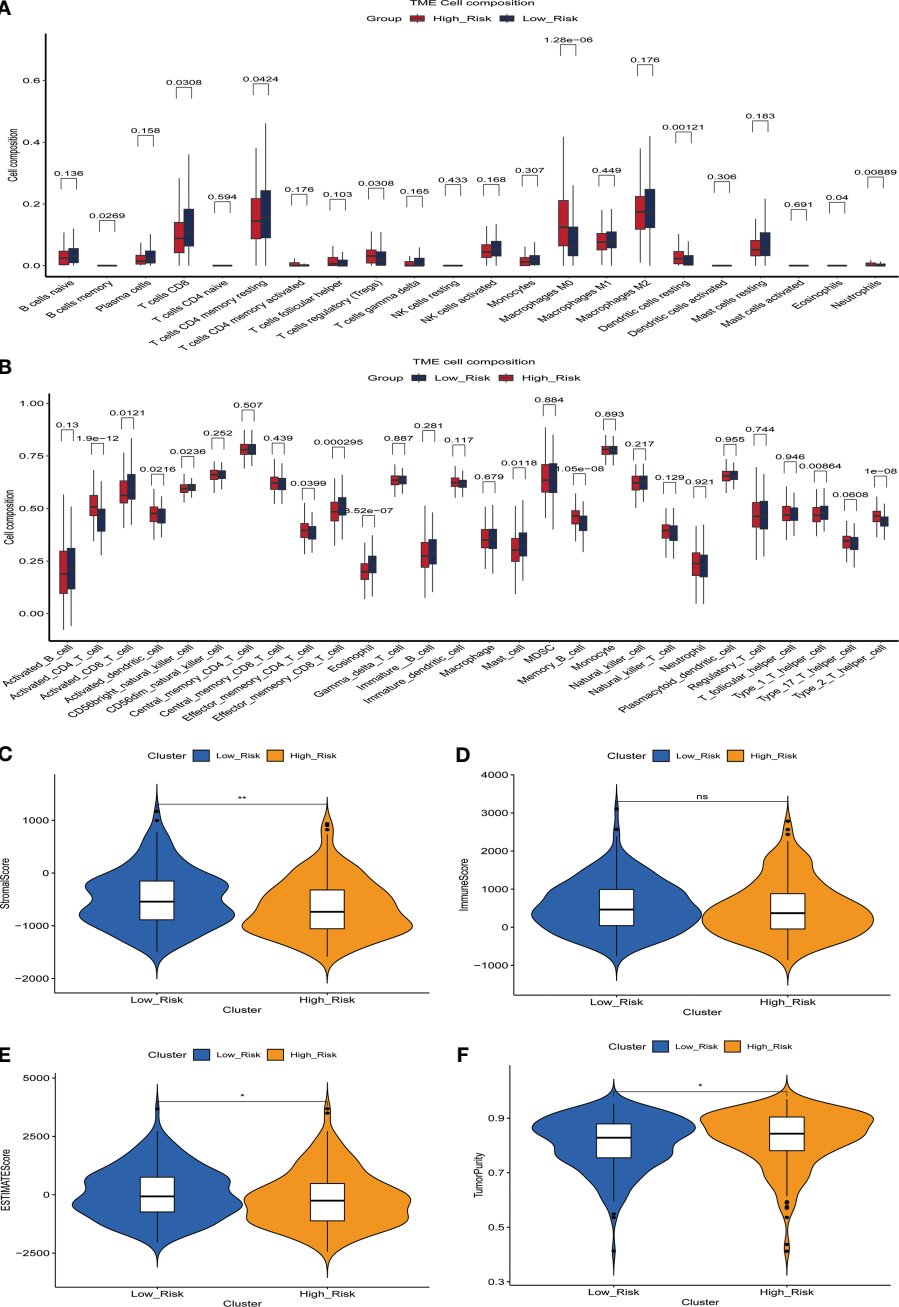
The relationship between prognostic signature and the proportion of immune cell infiltration in the tumor microenvironment. **(A)** Differences in the proportion of infiltrating immune cells between the high-risk and low-risk groups. **(B)** Differences in the proportion of 28 immune cell infiltrates in the tumor microenvironment between high- and low-risk groups. **(C-F)** Relationship between prognostic signature and StromalScore **(C)**, ImmuneScore **(D)**, ESTIMATEScore **(E)** and TumorPurity **(F)** in the training set.

The microenvironment of a tumor plays an essential role in the development and progression of the disease. We inferred the proportion of 22 immune cell types in the tumor microenvironment of 365 HCC patients using the CIBERSOR function of the R software. In addition, we evaluated the fraction of immune cells infiltrating the tumor microenvironment between high-risk and low-risk groups. In comparison to the low-risk group, the high-risk group had a higher proportion of immune cells with immunosuppressive functions, such as Tregs, Macrophages, Dendritic cells resting, and Neutrophils, and a lower proportion of immune cells with immunocidal functions, such as T cells CD8 and T cells CD4 memory resting (**Figure 5A**). To more comprehensively assess the differences in the infiltration of immune cells in the tumor microenvironment of 365 HCC patients, we used the GSVA function of R software to calculate the percentage of infiltration of 28 immune cell types. The results showed a higher proportion of Activated_CD4_T_cell, Activated_dendritic_cell, Memory_B_cell, and Type_2_T_helper_cell in the high-risk group compared to the low-risk group, while Activated_CD8_T_cell, CD56bright_natural_killer_cell, Effector_memeory_CD8_T_cell, and Type_1_T_helper_cell have a lower percentage(**Figure 5B**). These results suggest the presence of a immunosuppressive microenvironment in patients in the high-risk group, where the killer function of immune cells is suppressed, which partly explains the poorer prognosis of patients in the high-risk group.

We then evaluated the relationship between StromalScore, ImmuneScore, ESTIMATEScore, TumorPurity, and the ERGPS, and the results showed that ESTIMATEScore and StromalScore were higher in the low-risk group, whereas TumorPurity was higher in the high-risk group (**Figures 5C–F**).

### Relationship between the ERGPS and tumor mutation burden and microsatellite instability

**Figure 6:**
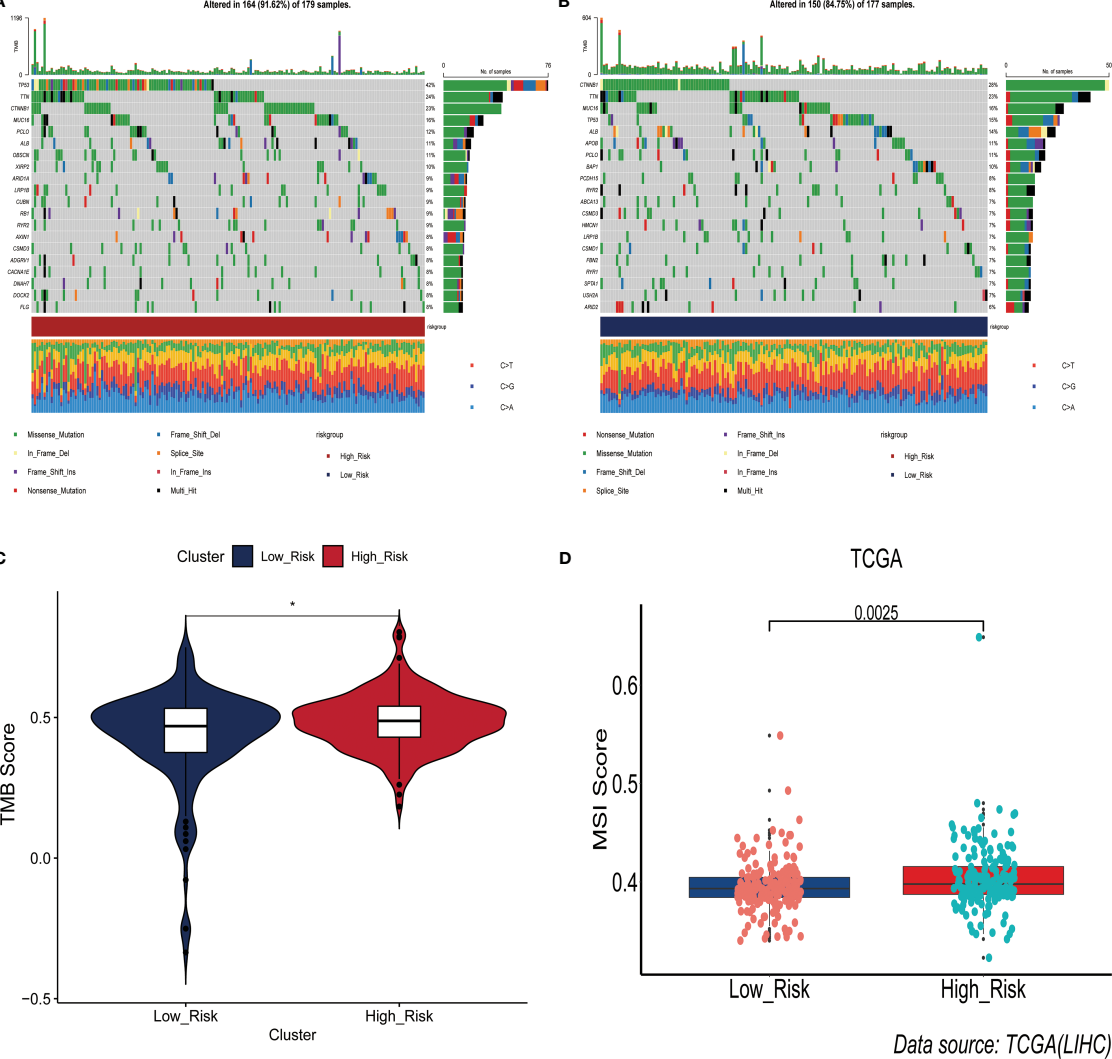
Association between prognostic signature and somatic mutations. **(A)** Landscape of somatic gene mutations in high-risk group of HCC patients. **(B)** Landscape of somatic gene mutations in low-risk group of HCC patients. **(C)** Differences between high- and low-risk groups in tumor mutation burden. **(D)** Differences in the distribution of microsatellite instability between the high- and low-risk groups.

Interestingly, we found that the frequency of somatic mutations in the high-risk group was 91.62%(**Figure 6A**), whereas in the low-risk group it was 84.75%(**Figure 6B**). TP53 was the gene with the highest mutation frequency in the high-risk group, whereas CTNNB1 had the highest mutation frequency in the low-risk group. The findings of a comparison of the difference in TMB scores between the two groups revealed that the high-risk group had a higher TMB score(p<0.05)(**Figure 6C**). Subsequently, we explored the relationship between ERGPS and MSI scores, and the results showed that the high-risk group had higher MSI scores compared with the low-risk group (p<0.05)(**Figure 6D**). These results suggest that the high frequency of TP53 gene mutations may be partly responsible for the poorer prognosis of HCC patients in the high-risk group. Meanwhile, patients in the high-risk group had a higher frequency of somatic mutations, a higher TMB score, and a higher MSI score, suggesting that patients in the high-risk group may be more responsive to immunotherapy and more suitable for immunotherapy.

### Prediction of immunotherapy response

**Figure 7:**
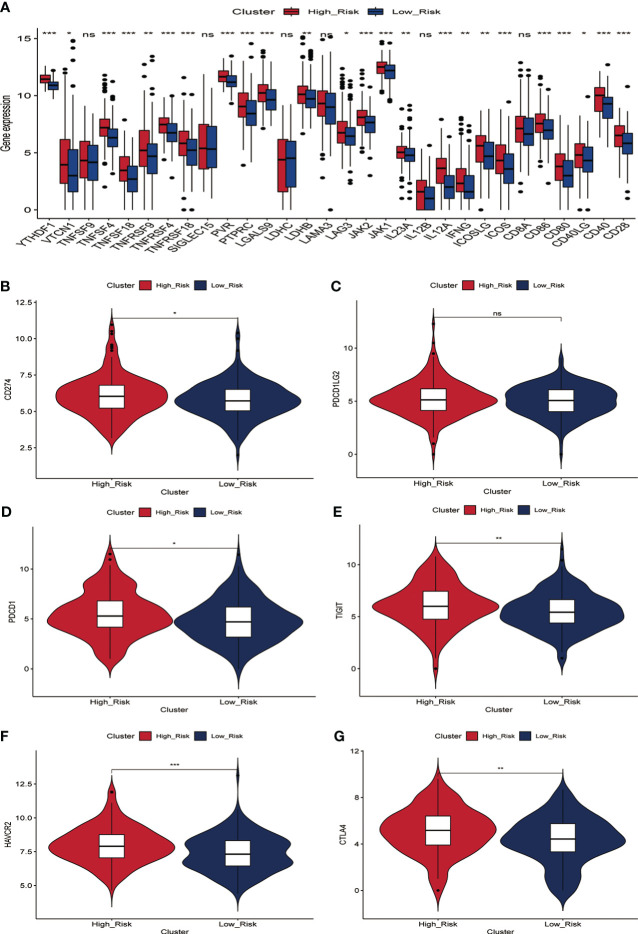
Relationship between prognostic signature and mRNA expression levels of immune checkpoint-associated genes in the training set. **(A)** The mRNA expression levels of most immune checkpoint-related genes were significantly higher in the high-risk group.**(B-G)** Differences in mRNA expression levels of six immune check-related genes commonly used in HCC immunotherapy between high- and low-risk groups, including PD-L1 **(B)**, PD-L2 **(C)**, PD1 **(D)**, TIGIT **(E)**, TIM-3 **(F)** and CTLA4 **(G)**.

**Figure 8:**
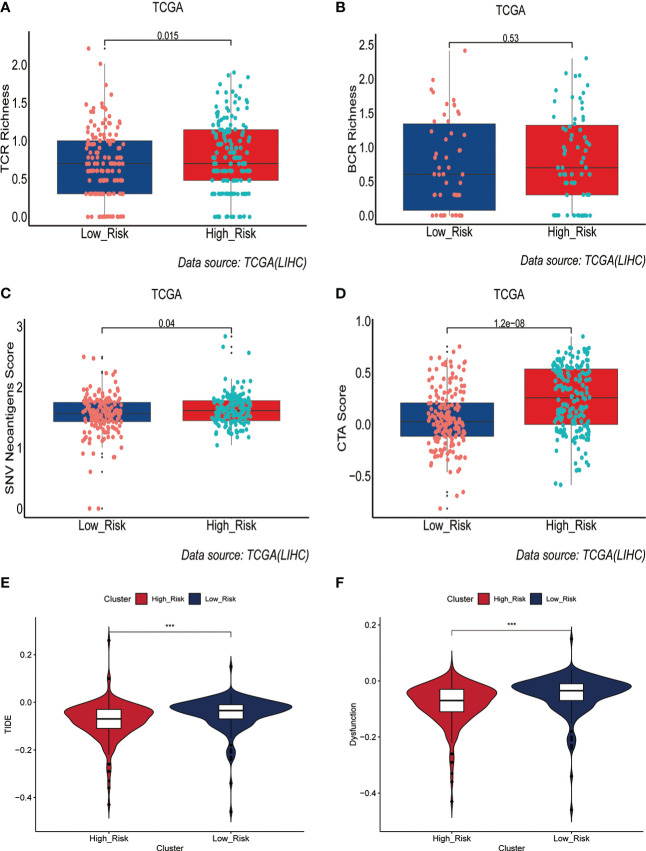
Prediction of immunotherapy response by prognostic signature in the training set. **(A-D)** Differences in TCR richness **(A)**, BCR richness **(B)**, SNV Neoantigens **(C)** and CTA scores **(D)** between the high-risk and low-risk groups. **(E-F)** TIDE scores **(E)** and Dysfunction scores **(F)** were used to assess immunotherapy response.

Immunotherapy acts against tumors by acting on tumor cells or immune checkpoint-associated proteins on the surface of immune cells. Therefore, the mRNA expression levels of immune checkpoint-related genes have an important role in the immunotherapy of tumors. We first compared the differences in mRNA expression levels of immune checkpoint-associated genes commonly used in most solid tumors between the two groups. The results showed that the mRNA expression levels of these immune checkpoint-associated genes were elevated in the high-risk group(**Figure 7A**, **Figure S7**). The relationship between ERGPS and mRNA expression levels of immune checkpoint-related genes (PD-L1, PDCD1LG2, PDCD1, TIGIT, TIM-3, CTLA4) commonly used in liver cancer immunotherapy was investigated further, and the results indicated that mRNA expression levels of these immune checkpoint-related genes were elevated in the high-risk group compared to the low-risk group (**Figures 7B–G**). These findings imply that immunosuppressive microenvironment exists in the high-risk group and that immunotherapy can reverse this immunosuppressive state so that patients can benefit. In previous research, T cell receptors (TCR) and B cell receptors (BCR) were responsible for recognizing antigens presented by MHC, and rearrangement analysis of TCR and BCR has proven to be an effective biomarker for stratifying and monitoring immunotherapy patients (19–22). To further evaluate the link between ERGPS and immunotherapy response, we evaluated the differences between the two groups’ TCR, BCR, SNV, and CTA scores. Intriguingly, we discovered that the high-risk group had higher TCR, BCR, SNV, and CTA scores than the low-risk group(**Figures 8A–D**), and these findings suggest that high-risk patients may be more susceptible to immunotherapy. The TIDE score, a recently discovered computational technique for modeling tumor immune evasion, is a more reliable biomarker for predicting immunotherapy response than TMB or PD-L1 expression (23). Further examination of the association between ERGPS and TIDE scores revealed that the TIDE scores and Dysfunction scores were considerably lower in the high-risk group compared to the low-risk group (**Figures 8E, F**). Since patients with a lower TIDE score are more likely to have a lesser chance of antitumor immune escape, immune checkpoint blockade (ICB) treatment has a better response rate (24). These data imply that immunotherapy was more effective for HCC patients with higher risk scores in the training set.

### Independent external immunotherapy data for validation

**Figure 9:**
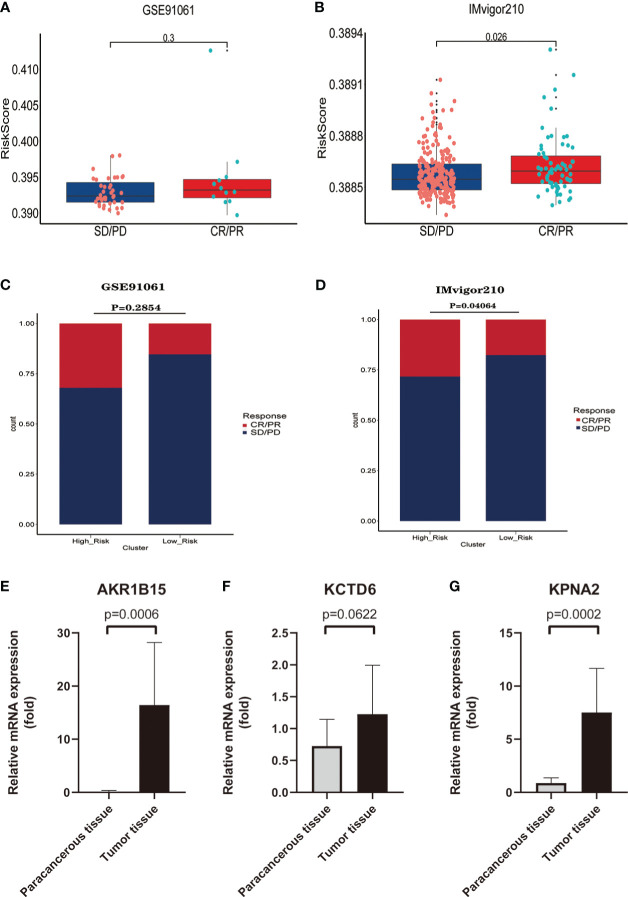
Independent external immunotherapy data and RT-qPCR techniques were used to validate the validity and robustness of the prognostic signature. **(A)** In the GSE91061 dataset, patients who responded to immunotherapy (CR/PR) had a higher median risk score than patients in the low-risk group.**(B)** In the IMvigor210 dataset, patients who responded to immunotherapy (CR/PR) had significantly higher risk scores than patients in the low-risk group.**(C)** In the GSE91061 dataset, the percentage of patients who responded to immunotherapy (CR/PR) was higher in the high-risk group than in the low-risk group. **(D)** In the IMvigor210 dataset, the percentage of patients who responded to immunotherapy (CR/PR) was significantly higher in the high-risk group than in the low-risk group.**(E-G)** qRT-PCR results confirmed that the three estrogen-related genes used to construct the prognostic signature were overexpressed in HCC tissues, including AKR1B15 **(E)**,KCTD6 **(F)** and KPNA2 **(G)**.

To further explore the value of ERGPS in predicting immunotherapy response, two independent immunotherapy data (IMvigor210, GSE91061) were included in the study. The IMvigor210 dataset has 298 patients treated with anti-PD-L1 and the GSE91061 dataset contains 51 patients treated with anti-PD-L1 or Anti-CTLA4; both datasets include entire transcriptome sequencing data and matched clinical information. We calculated risk scores for each patient in both immunotherapy datasets using the same formula as in the training set, and divided patients receiving immunotherapy into low- and high-risk groups based on the median value of the risk scores. Intriguingly, we discovered a link between risk scores and immunotherapy response, with higher risk scores among patients with CR/PR than SD/PD (**Figures 9A, B**). Comparing the proportion of patients with CR/PR between the high-risk and low-risk groups, we determined that the proportion of patients with CR/PR was greater in the high-risk group than in the low-risk group in both datasets (**Figures 9C, D**). These validate the important value of ERGPS in predicting immunotherapy response in oncology patients.

### Analysis of the correlation between ERGPS and common chemotherapeutics

To evaluate ERGPS in the clinic for HCC treatment, we attempted to explore associations between risk scores and the efficacy of administering common chemotherapeutics. A greater risk score was associated with a lower IC50 for anticancer medicines such as Midostaurin, Salubrinal, Tipifarnib, Etoposide,Embelin, and Doxorubicin, according to our study(**Figure S8**). These results suggest that patients in the high-risk category may be more responsive to these anticancer drugs than patients in the low-risk group.

### qRT-PCR confirmed overexpression of three estrogen-related gene in HCC tissues

To further validate the robustness of the prognostic signature, we collected tumor tissue samples and matched paraneoplastic samples from 12 patients with HCC confirmed by postoperative pathology. qRT-PCR results confirmed that the mRNA expression levels of all these three estrogen-related genes(AKR1B15, KCTD6, KPNA2) in ERGPS were significantly higher in HCC tissue than in paraneoplastic tissue(**Figures 9E–G**). These results confirmed the robustness of ERGPS.

## Discussion

In recent years, tyrosine kinase inhibitors (TKI), exemplified by sorafenib, have been able to extend the survival of a subset of HCC patients, but systemic therapy still confronts numerous obstacles (25, 26). With the unveiling of clinical studies of programmed death receptor 1 (PD-1) and cytotoxic T-lymphocyte-associated protein 4 (CTLA4) inhibitors, immune checkpoint inhibitor (ICI)-based immunotherapy has shown encouraging efficacy in multiple cancer types (27–29). Despite significant advancements in the use of immunotherapy for HCC, only a small percentage of patients benefit from it due to factors such as decreased tumor immunogenicity, antigen-presenting mutations, missing mutations in signaling pathways, and decreased immune cell function in the tumor microenvironment (2, 30, 31). Some immunotherapy recipients exhibit no response or significant immune-related side effects, and a small number of immunotherapy recipients even develop HPD (32, 33). Therefore, there is an urgent need for effectively predictive biomarkers to assist in screening potential populations that could benefit from immunotherapy, further to guide the rational application of immune drugs in clinical practice, improve patient response rates, and reduce the risk of immunotoxicity in patients.

As a potent endogenous antioxidant, estrogen contributes to the development of a variety of liver disorders, including liver fibrosis, NAFLD, and hepatocellular carcinoma (34–36). In addition to being a consequence of disease progression, abnormal estrogen levels may also play a role in the pathophysiology of the development of chronic liver disorders. Also, estrogen has non-reproductive effects as a regulator of the immune system, growth, neurological function, and metabolism. In particular, impaired expression and function of estrogen receptors in the liver are closely associated with obesity and liver-related metabolic dysfunction (37). A growing number of studies have established that estrogen signaling plays an immunosuppressive role in the tumor microenvironment, hence promoting tumor cell migration and invasion (38–40). Chronic liver disease and liver cancer are more prevalent in men (41), while men with cirrhosis and HCC have increased serum estrogen levels (42, 43). In addition, serum estrogen levels are elevated in patients undergoing surgical hepatectomy, suggesting the importance of estrogen regulation in the liver regeneration process. The mechanisms by which the liver senses and responds to estrogen to affect liver growth and cancer formation remain undetermined. In this study, through a comprehensive analysis of transcriptome data from 365 HCC patients, we developed a novel estrogen-related gene prognostic signature (ERGPS) in the current study. To study the association between estrogen-related genes and immunotherapy, we undertook a complete profiling in terms of mRNA expression levels of immune cell infiltration, somatic mutations, TMB, MSI, TCR, BCR, SNV, TIDE, and immune checkpoint-related genes. In the microenvironment of HCC tumors, estrogen promotes the infiltration of immune cells with immunosuppressive functions while rejecting the invasion of immune cells with killing functions. In the meantime, the expression of estrogen-related genes may increase the mRNA expression of immune checkpoint-related genes. These findings indicate that estrogen has a significant role in tumor cell growth, invasion, and immune cell evasion. Consequently, estrogen-related genes have the potential to serve as new immunotherapeutic targets in HCC.

In this study, ERGPS includes three estrogen-related genes (AKR1B15, KCTD6, and KPNA2), all of which are connected with the prognosis of HCC patients and are involved in estrogen production. AKR1B15 (Aldo-Keto Reductase Family 1 Member B15) is a Protein Coding gene (44). Its related pathways are Metabolism and Metabolism of steroid hormones. Studies have shown that AKR1B10 can induce a variety of cancers, such as HCC (45), non-small cell lung cancer (46), and pancreatic cancer (47), and is a promising potential cancer target. KCTD6 (Potassium Channel Tetramerization Domain Containing 6) is a Protein Coding gene. Diseases associated with KCTD6 include Medulloblastoma. Among its related pathways are Sweet Taste Signaling and Class I MHC-mediated antigen processing and presentation (48). There are currently few investigations on the correlation between KCTD6 and cancer. We validated KCTD6 overexpression in clinical samples using RT-PCR, and survival analysis revealed that the overall survival time of HCC patients in the overexpression KCTD6 group was significantly worse than in the low expression group, indicating that KCTD6 overexpression may promote tumor progression in HCC. Karyopherin α2 (KPNA2) belongs to the karyopherin family, which plays a crucial role in nucleocytoplasmic transport (49). Guo et al (50) verified that the overexpression of KPNA2 can accelerate the proliferation and migration of tumor cells and is related to a poor prognosis in patients with HCC. These findings demonstrate that overexpression of estrogen-related genes can increase tumor cell proliferation and invasion, and that these genes are prospective immunotherapy targets for HCC.

In this study, the prognosis signature proved to be a powerful prediction tool for the prognosis of HCC patients in the training set and verification set. The outstanding predicted accuracy of the prognostic signature motivated us to investigate the underlying molecular pathways. Initially, we did GO and KEGG analyses to investigate the enrichment pathways of genes closely connected with ERGPS. The results indicated that these genes were primarily enriched in the CELL CYCLE, OOCYTE MEIOSIS signaling pathway. Therefore, the poorer prognosis of individuals with high-risk scores may be partially attributable to the aberrant regulation of the cell cycle, which is closely linked to tumor proliferation and progression. Tumor-infiltrating immune cells play an important role in the tumor microenvironment and greatly influence the prognosis of HCC patients. We used the CIBERSORT algorithm of the R software to calculate the proportion of immune cells infiltrating the tumor microenvironment in the training set for each patient. Interestingly, we found that the prognostic signature was positively correlated with the proportion of cells with immunosuppressive functions, such as Treg cells, dendritic cells, and macrophages. These findings indicate that overexpression of estrogen-related genes may recruit these cells to the tumor microenvironment, thereby exerting an immunosuppressive impact and allowing tumor cells to evade immune cell surveillance. In conclusion, with all the above findings, we infer that the powerful predictive power of ERGPS may lie in cell cycle dysregulation and immunosuppressive microenvironment. Therefore, inhibition of estrogen-related gene overexpression may inhibit the convergence of immunosuppressive immune cells to the tumor microenvironment and thus exert an anti-tumor effect.

The success of immunotherapy for HCC is related to many factors, such as the immunogenicity of tumor cells, mRNA expression levels of immune checkpoint-related genes, and the functionality of tumor-infiltrating T cells. The mRNA expression level of immune checkpoint-related genes is a well-recognized biological marker for determining the response to immunotherapy. We first explored the relationship between the mRNA expression levels of immune checkpoint-related genes and ERGPS, and interestingly, we found that ERGPS was positively correlated with the mRNA expression levels of most immune checkpoint-related genes. The mRNA expression levels of immune checkpoint-related genes were significantly higher in the high-risk group than in the low-risk group, especially for immune checkpoint-related genes commonly used in liver cancer immunotherapy, including (PD-L1, PDCD1LG2, PDCD1, TIGIT, TIM-3, CTLA4). These results further suggest that immune cells in the tumor microenvironment of patients in the high-risk group form an immunosuppressive microenvironment through immune checkpoint interactions with immunomodulatory cells with immunosuppressive functions, allowing tumor cells to evade the immune system; immunotherapy can block this interaction to reverse immunosuppressive state. Patients in the high-risk group are consequently more likely to respond to immunotherapy than those in the low-risk group. TMB and MSI have been demonstrated to be valuable tools for predicting immunotherapy response in a range of solid cancers. In this study, we investigated the relationship between ERGPS, TMB, and MSI. The results indicated that ERGPS was positively correlated with TMB and MSI, and that the TMB and MSI scores in the high-risk group were significantly higher than those in the low-risk group, indicating that tumor cells in the high-risk group had a higher immunogenicity. SNV and CTA levels can partially reflect the immunogenicity of tumor cells. In this study, patients in the high-risk group had higher SNV and CTA scores than those in the low-risk group, indicating that the high-risk group’s tumor cells were more immunogenic. TCR and BCR are unique molecules on the surface of T and B cells, respectively, which recognize MHC-presented antigens. Studies of TCR, and BCR profiles in multiple cancer types have shown that TCR and BCR profiles can be used as predictive biomarkers of response to treatment with CTLA-4 or PD-1 inhibitors (19, 20, 22). We evaluated the differences in TCR richness and BCR richness between the high-risk and low-risk groups and found that the high-risk group had significantly higher TCR abundance and BCR richness than the low-risk group, which reflected greater functionality of T cells and B cells in recognizing antigens and killing tumor cells in high-risk HCC patients. In addition, the TIDE prediction score can predict the effectiveness of immunotherapy for a wide range of cancers. In our study, we observed that patients in the high-risk group had lower TIDE scores compared to the low-risk group, suggesting that patients with HCC in the high-risk group had a better response to immunotherapy. To further validate our conclusions, we used two independent external immunotherapy data (Imvigor210, GSE91061) for validation. The results revealed a larger proportion of immunotherapy responders in the high-risk group, indicating that high-risk patients are more likely to benefit from immunotherapy. With more confirmation, ERGPS has the potential to serve as a reliable biomarker for predicting immunotherapy response in HCC.

Despite the encouraging results, the study has some limitations. First, our study was conducted based on a public database and lacks validated experiments to validate the molecular mechanisms behind the prognostic signature. Secondly, the validity of our prognostic siganture was not validated with a large number of clinical samples. Finally, all the mechanistic analyses in this study are descriptive in this study. A large number of future studies are needed for validation.

In conclusion, a three-gene signature based on estrogen-related genes was identified and validated to have powerful performance in predicting prognosis and immunotherapy in HCC patients. It may provide a deeper understanding and new insights into developing novel immunotherapies for HCC. Also, it can be used as a prognostic biomarker for individualized prediction of clinical decisions and to help screen appropriate patients who may benefit from immunotherapy.

## Data availability statement

The original contributions presented in the study are included in the article/**Supplementary Material**. Further inquiries can be directed to the corresponding authors.

## Author contributions

BG and YW performed the data analysis and wrote the manuscript. These authors contributed equally to this work. CL and SL reviewed and revised the manuscript. These corresponding authors contributed equally to this work. All authors contributed to the article and approved the submitted version.

## Supplementary material

Supplementary Figure 1 Flowchart of the data analysis procedures.

Supplementary Figure 2 The relationship between risk scores and clinical characteristics in the training set. **(A–H)** Relationship between risk score and clinical characteristics, including age **(A)**, gender **(B)**, N stage **(C)**, M stage **(D)**, tumor grade **(E)**, tumor stage**(F)**, MVI**(G)** and survival status **(H)**.

Supplementary Figure 3 Validation of prognostic signature in clinical subgroups. **(A-F)** The validity of the prognostic signature was validated in different clinical subgroups, including young **(A)**, old **(B)**, male **(C)**, female **(D)**, MVI negative **(E)** and MVI positive **(F)**.

Supplementary Figure 4 The predictive efficacy of prognostic signature was verified in different clinical subgroups. **(A-D)** Kaplan-Meier curves showed that in different clinical subgroups, such as low tumor grade **(A)**, high tumor grade **(B)**, early stage **(C)** and advanced stage **(D)**, the overall survival time of low-risk group was significantly better than that of high-risk group.

Supplementary Figure 5 Exploration of the molecular mechanisms behind the prognostic signature. **(A)** The volcano map demonstrates that most genes are highly expressed in the high-risk group. **(B)** Results of GO functional enrichment analysis of genes highly expressed in the high-risk group. **(C)** Results of GO functional enrichment analysis of genes highly expressed in the low-risk group. **(D)** Results of GSEA KEGG functional enrichment analysis in the high-risk and low-risk groups.

Supplementary Figure 6 Results of HALLMARKER pathway enrichment analysis in the high-risk and low-risk groups. **(A-C)** The enrichment results of HALLMARKER pathway showed that E2F_TARGETS **(A)**, G2M_CHECKPOINT **(B)** and DNA_REPAIR **(C)** were mainly enriched in high-risk group, while COAGULATION **(D)** was mainly enriched in low-risk group

Supplementary Figure 7 Heatmap shows that most immune checkpoint-associated genes are overexpressed in high-risk groups.

Supplementary Figure 8 Drug sensitivity prediction in the training set. **(A-F)** The results of drug sensitivity analysis showed a higher risk score was related to lower IC50 among antitumor drugs, such as Midostaurin **(A)**, Salubrinal **(B)**, Tipifarnib **(C)**, Etoposide **(D)**,Embelin **(E)** and Doxorubicin **(F)**.
