# Supplementary figures and images for: Estrogen-related genes influence immune cell infiltration and immunotherapy response in Hepatocellular Carcinoma
Source: Front Immunol. 2023 Feb 6;14:1114717. doi: 10.3389/fimmu.2023.1114717 (PMC9939443; doi:10.3389/fimmu.2023.1114717)

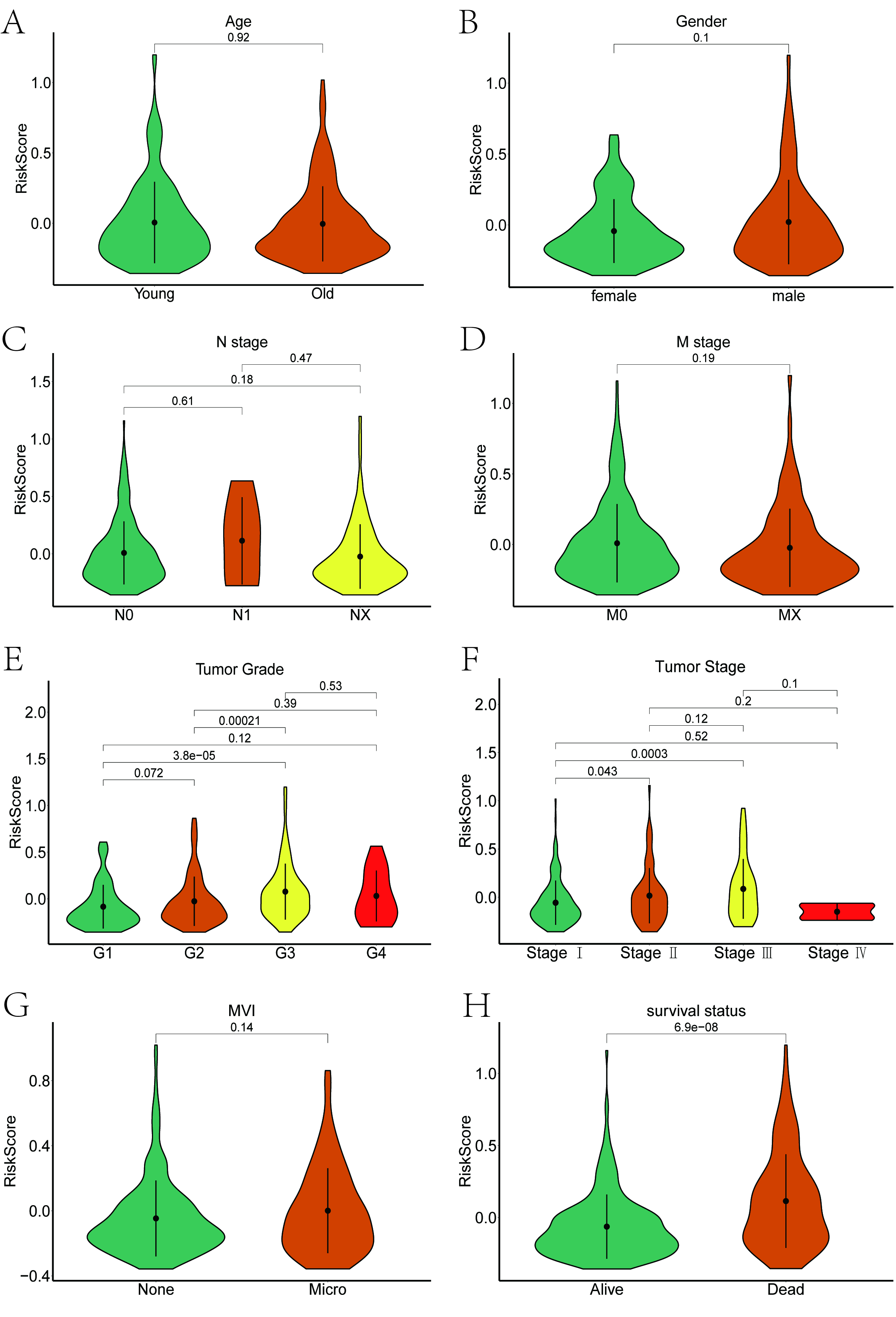

Supplement: Supplementary Figure 2 — The relationship between risk scores and clinical characteristics in the training set. (A–H) Relationship between risk score and clinical characteristics, including age (A), gender (B), N stage (C), M stage (D), tumor grade (E), tumor stage(F), MVI(G) and survival status (H). [file Image_2.tif]

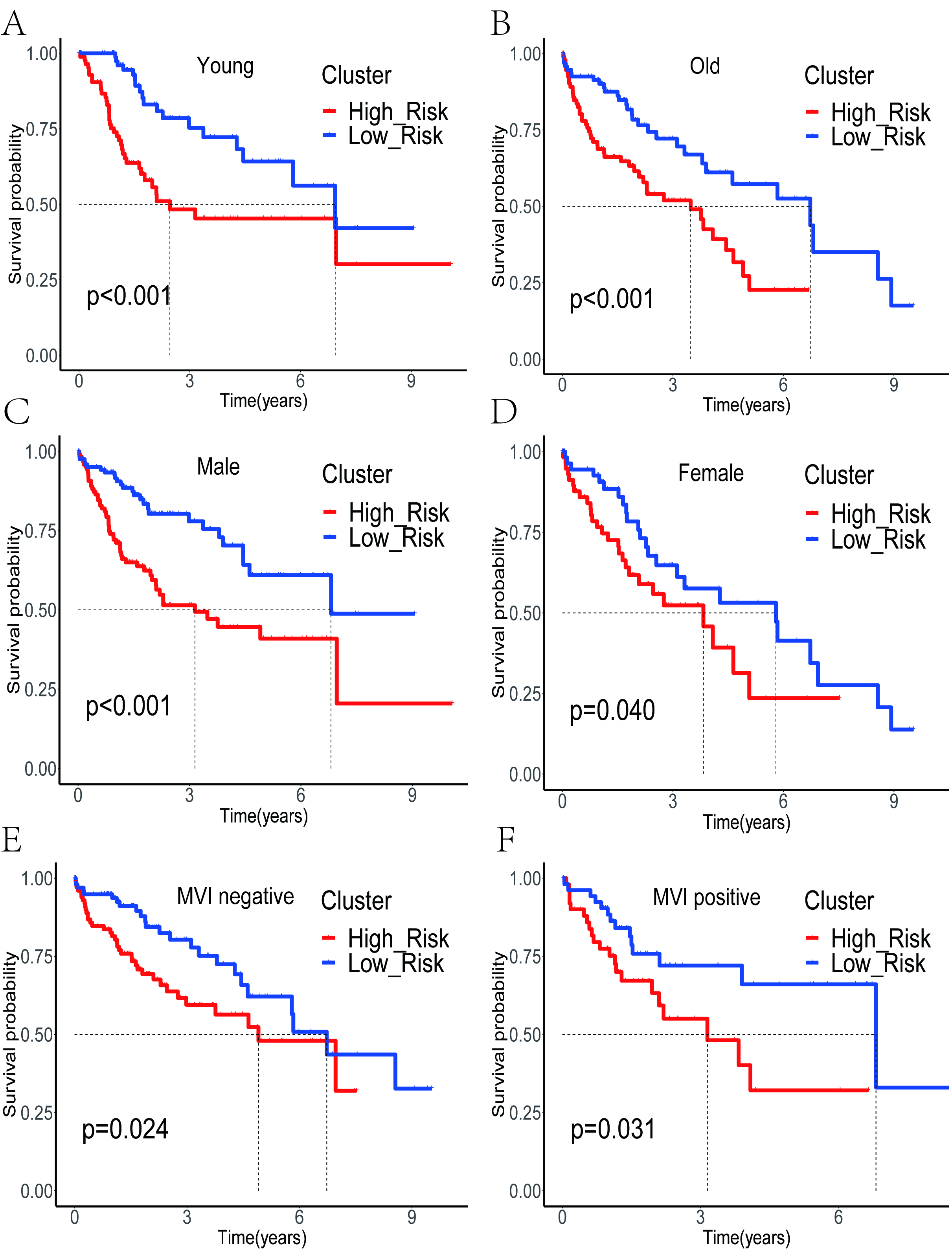

Supplement: Supplementary Figure 3 — Validation of prognostic signature in clinical subgroups. (A-F) The validity of the prognostic signature was validated in different clinical subgroups, including young (A), old (B), male (C), female (D), MVI negative (E) and MVI positive (F). [file Image_3.tif]

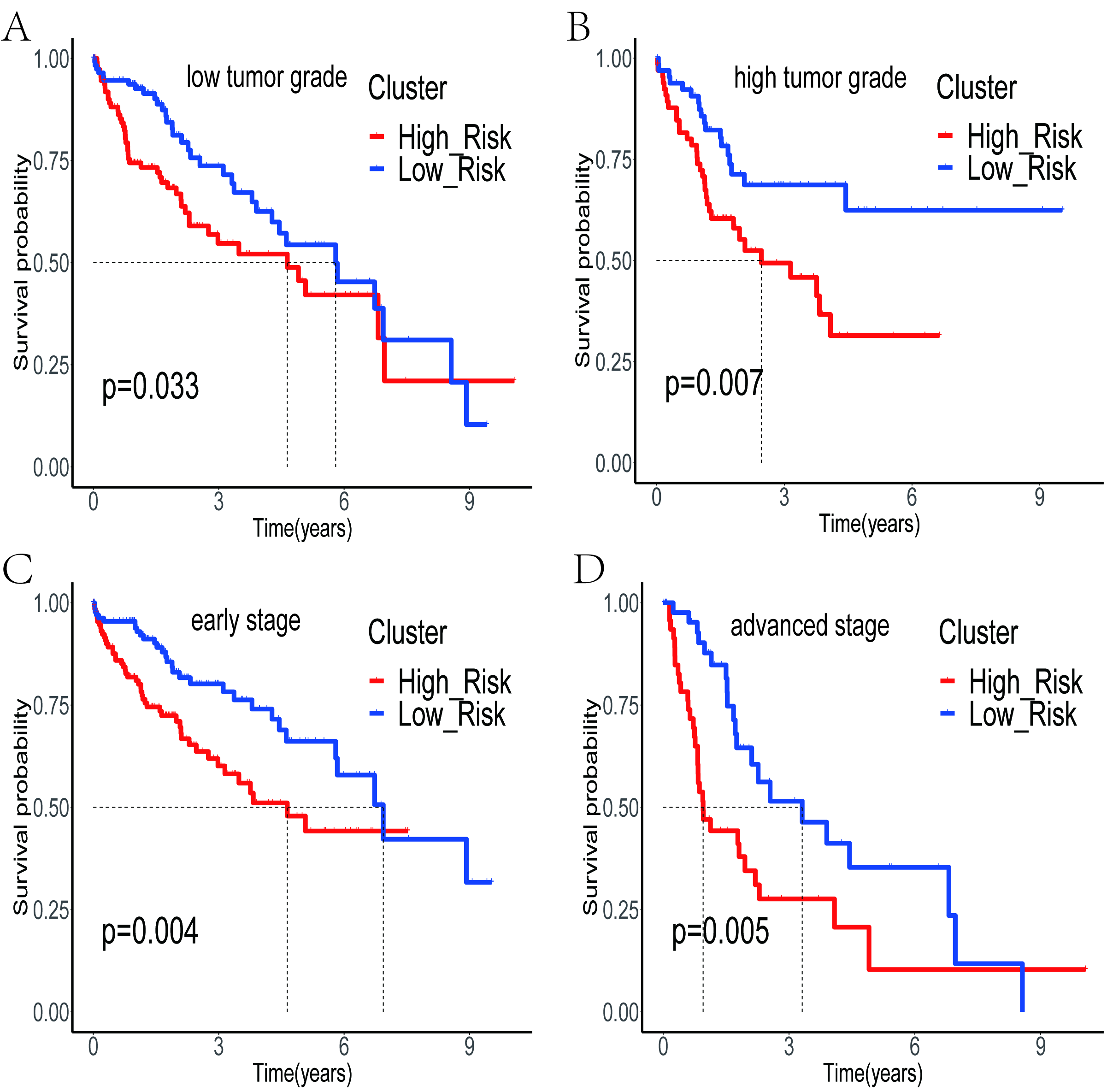

Supplement: Supplementary Figure 4 — The predictive efficacy of prognostic signature was verified in different clinical subgroups. (A-D) Kaplan-Meier curves showed that in different clinical subgroups, such as low tumor grade (A), high tumor grade (B), early stage (C) and advanced stage (D), the overall survival time of low-risk group was significantly better than that of high-risk group. [file Image_4.tif]

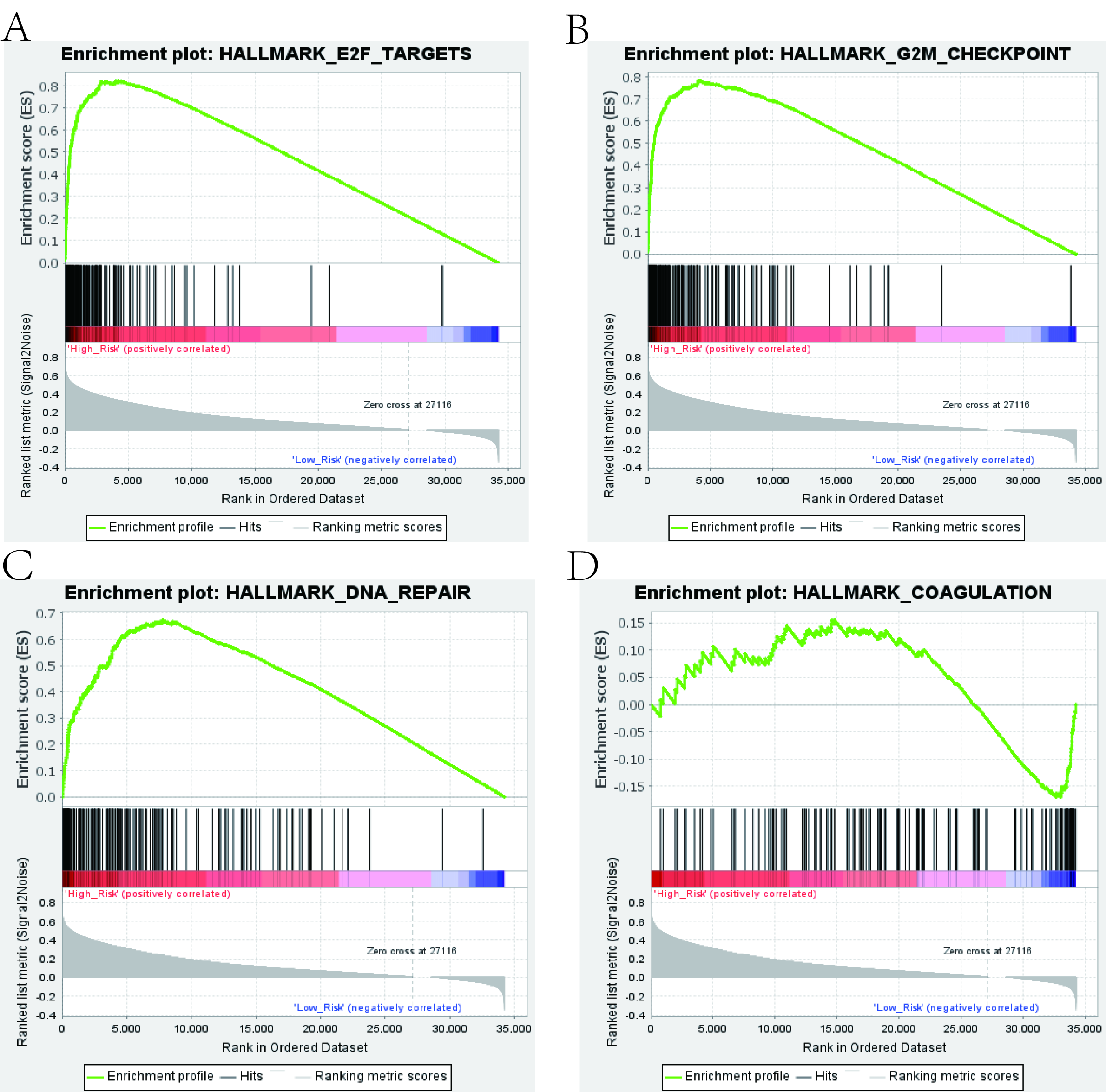

Supplement: Supplementary Figure 6 — Results of HALLMARKER pathway enrichment analysis in the high-risk and low-risk groups. (A-C) The enrichment results of HALLMARKER pathway showed that E2F_TARGETS (A), G2M_CHECKPOINT (B) and DNA_REPAIR (C) were mainly enriched in high-risk group, while COAGULATION (D) was mainly enriched in low-risk group [file Image_6.tif]

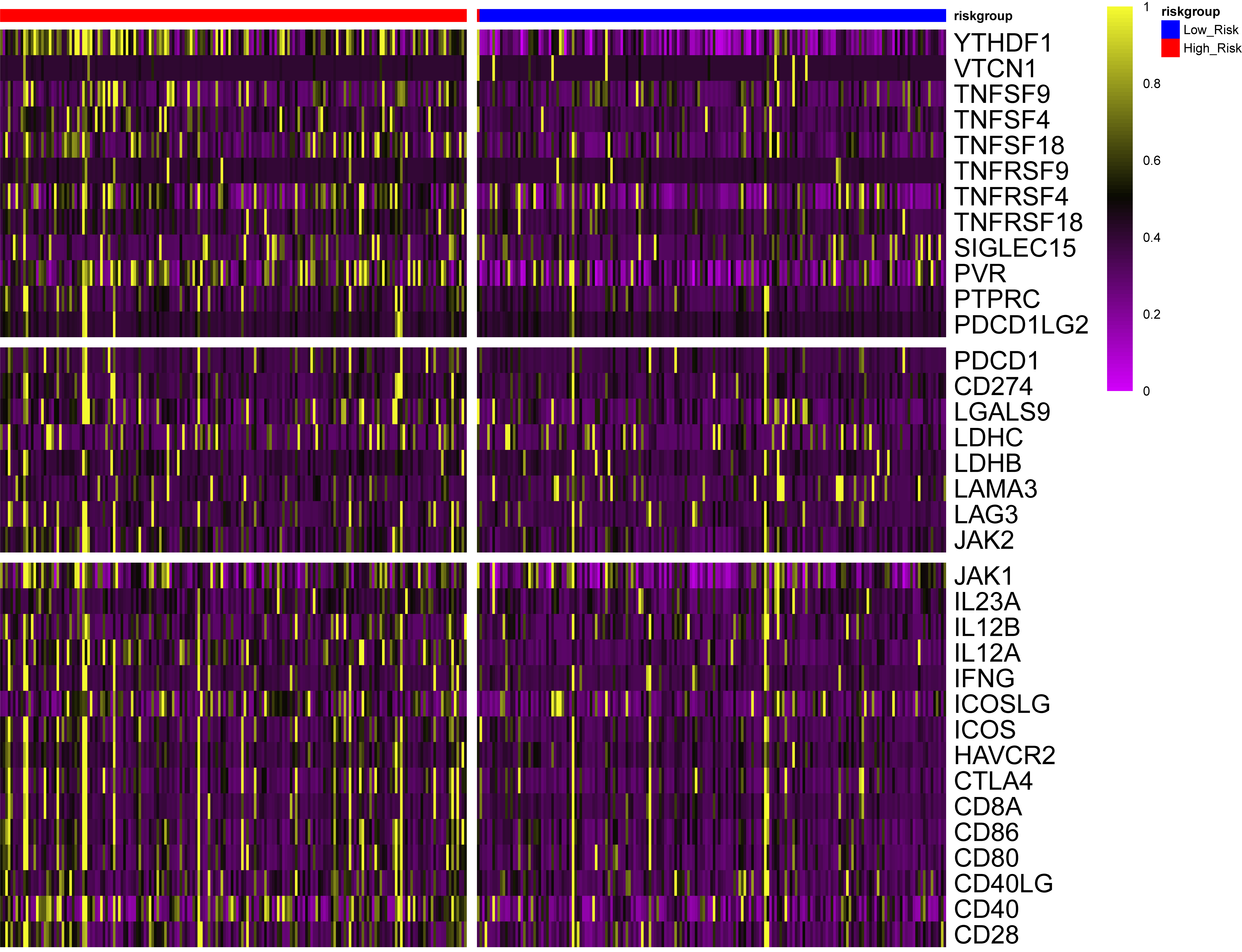

Supplement: Supplementary Figure 7 — Heatmap shows that most immune checkpoint-associated genes are overexpressed in high-risk groups. [file Image_7.tif]

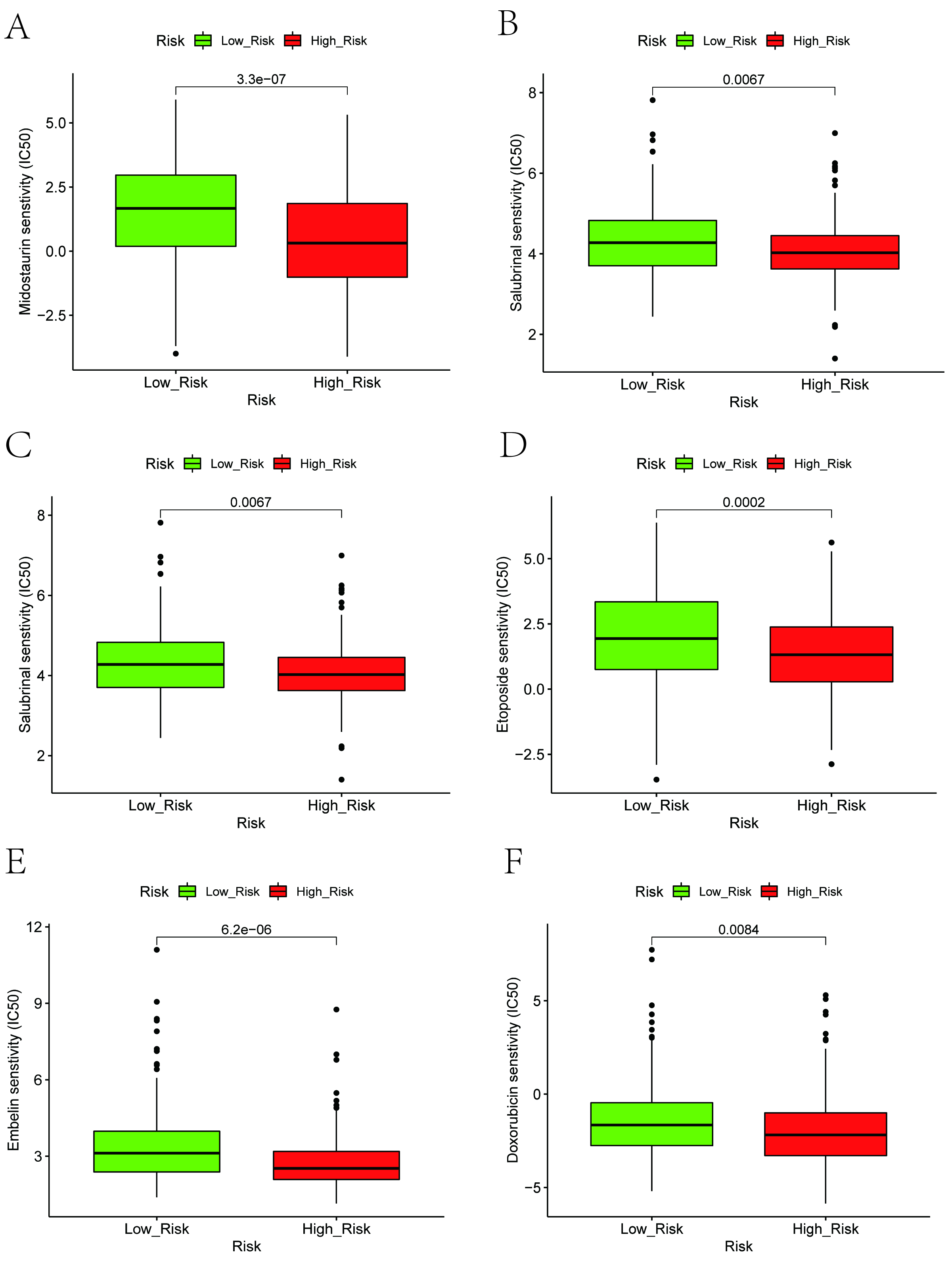

Supplement: Supplementary Figure 8 — Drug sensitivity prediction in the training set. (A-F) The results of drug sensitivity analysis showed a higher risk score was related to lower IC50 among antitumor drugs, such as Midostaurin (A), Salubrinal (B), Tipifarnib (C), Etoposide (D),Embelin (E) and Doxorubicin (F). [file Image_8.tif]
